# The LIM-domain only protein 4 contributes to lung epithelial cell proliferation but is not essential for tumor progression

**DOI:** 10.1186/s12931-015-0228-0

**Published:** 2015-06-07

**Authors:** Aliaksei Z. Holik, Caitlin E. Filby, Julie Pasquet, Kati Viitaniemi, John Ciciulla, Kate D. Sutherland, Marie-Liesse Asselin-Labat

**Affiliations:** ACRF Stem Cells and Cancer Division, The Walter and Eliza Hall Institute of Medical Research, 1G Royal Parade, Parkville, VIC 3052 Australia; Department of Medical Biology, The University of Melbourne, Parkville, VIC Australia; The Alfred Hospital, Melbourne, VIC Australia

**Keywords:** Lung injury, Progenitor cells, Lung cancer, LMO4

## Abstract

**Background:**

The lung is constantly exposed to environmental challenges and must rapidly respond to external insults. Mechanisms involved in the repair of the damaged lung involve expansion of different epithelial cells to repopulate the injured cellular compartment. However, factors regulating cell proliferation following lung injury remain poorly understood. Here we studied the role of the transcriptional regulator Lmo4 during lung development, in the regulation of adult lung epithelial cell proliferation following lung damage and in the context of oncogenic transformation.

**Methods:**

To study the role of Lmo4 in embryonic lung development, lung repair and tumorigenesis, we used conditional knock-out mice to delete Lmo4 in lung epithelial cells from the first stages of lung development. The role of Lmo4 in lung repair was evaluated using two experimental models of lung damage involving chemical and viral injury. The role of Lmo4 in lung tumorigenesis was measured using a mouse model of lung adenocarcinoma in which the oncogenic K-Ras protein has been knocked into the K-Ras locus. Overall survival difference between genotypes was tested by log rank test. Difference between means was tested using one-way ANOVA after assuring that assumptions of normality and equality of variance were satisfied.

**Results:**

We found that Lmo4 was not required for normal embryonic lung morphogenesis. In the adult lung, loss of Lmo4 reduced epithelial cell proliferation and delayed repair of the lung following naphthalene or flu-mediated injury, suggesting that Lmo4 participates in the regulation of epithelial cell expansion in response to cellular damage. In the context of K-Ras^G12D^-driven lung tumor formation, Lmo4 loss did not alter overall survival but delayed initiation of lung hyperplasia in K-Ras^G12D^ mice sensitized by naphthalene injury. Finally, we evaluated the expression of LMO4 in tissue microarrays of early stage non-small cell lung cancer and observed that LMO4 is more highly expressed in lung squamous cell carcinoma compared to adenocarcinoma.

**Conclusions:**

Together these results show that the transcriptional regulator Lmo4 participates in the regulation of lung epithelial cell proliferation in the context of injury and oncogenic transformation but that Lmo4 depletion is not sufficient to prevent lung repair or tumour formation.

**Electronic supplementary material:**

The online version of this article (doi:10.1186/s12931-015-0228-0) contains supplementary material, which is available to authorized users.

## Background

The regulator of transcription LMO4 is a member of the LIM-domain only (LMO) protein family that act as molecular adaptors, providing a scaffold for multiprotein complexes of DNA-binding factors and transcriptional regulatory proteins [1–4]. LMO4 modulates neuronal progenitor cell proliferation and inhibits differentiation of mammary epithelial cells, suggesting that it may participate in the regulation of progenitor cell function [2, 5]. LMO4 is highly expressed in epithelial cells, often at location of active mesenchymal-epithelial interactions, such as hair follicles, teeth, epidermis, mammary gland, kidney, and lung [6–9] suggesting that LMO4 may modulate the function of DNA-binding proteins in signaling pathways involved in mesenchymal-epithelial cross-talk. LMO4 interacts with Smad proteins and increases the effect of TGFβ on epithelial cell growth [10]. Mice lacking Lmo4 die perinatally from complex phenotypic abnormalities, with approximately 50 % of mice exhibiting exencephaly. Mice that do not display exencephaly are pale at birth, gasping for air and die shortly after birth [11]. Deregulation of LMO4 has been described in several tumor types including breast, prostate, pancreas, lung and squamous cell carcinoma of the oral cavity [2, 12–16]. LMO4 is overexpressed in more than 50 % of breast cancers and high nuclear expression of LMO4 has been found to be a predictor of worse outcome in breast cancer patients [3]. Down-regulation of LMO4 reduced proliferation of human breast cancer cells and increased differentiation of mouse mammary epithelial cells while overexpression of LMO4 in non-invasive, immortalised human cells promoted cell motility and invasion [3], suggesting that Lmo4 may play a role in epithelial cell proliferation and differentiation.

The lung is constantly exposed to toxic agents and pathogens present in the environment and must activate a number of molecular and cellular processes to induce rapid cell proliferation and repair. It is becoming evident that distinct types of lung stem/progenitor cells are activated depending on the type of injury inflicted to the lung [17]. Experimental protocols using chemical agents, viruses, or diffusible gases, have been used in mouse models to study the activation of lung stem cells in response to environmental challenges. Each model is unique in the type of injury caused as reflected by the degree of immune cell infiltration, fibrosis and the cell types affected [18, 19]. Specifically, administration of H1N1 flu virus to animals leads to broad damage of epithelial cell types in the lung including club cells, ciliated cells, as well as alveolar cells. Damage and repair of the lung after H1N1 infection occurs in the absence of fibrosis as opposed to bleomycin treatment that induces extensive fibrosis [20]. A rare population of distal lung basal progenitor cells with regenerative capacity has been identified and shown to be responsible for the repair of the lung after H1N1 injury. Lineage tracing experiments and transplantation assays demonstrated that these rare distal basal cells expanded after injury and gave rise to club cells, ciliated cells and alveolar cells [21, 22]. Vaughan *et al.* indicated that these progenitor cells did not express lung lineage specific markers but were present in a subset of cells expressing integrin β4 (CD104) but negative for the club cell marker CC10 [22]. In the non-injured lung, cells expressing the laminin receptor integrin α6β4 were shown to be enriched for cells with colony forming capacity *in vitro* [23, 24], suggesting they behaved as progenitor cells in the distal lung. Chemical injury induced by administration of naphthalene, a component found in tobacco smoke, leads to the ablation of the large majority of club cells (Cyp2f2^+^). Only a small number of these cells, named variant club cells, that do not express Cyp2f2 resist injury and are thought to be the progenitor cells responsible for repair of the airways [25, 26]. Naphthalene injury has also been shown to accelerate tumour growth when combined with oncogenic alterations such as expression of K-Ras^G12D^ [27]. Factors involved in the regulation of the different classes of progenitor cells in the lung remain poorly characterised.

The observation that Lmo4 knockout mice display breathing difficulty at birth and that LMO4 is overexpressed in advanced lung cancer prompted us to explore its role in lung morphogenesis, adult lung repair and cancer. We used conditional knock-out mice to ablate Lmo4 expression in the lung epithelium from E9.5 and found that *Shh-cre;Lmo4*^*fl/fl*^ mice were viable and healthy. However, we observed that Lmo4 loss reduced proliferation of adult lung epithelial cells and delayed repair following virus-induced and chemical-induced lung injury. We then examined the role of Lmo4 in lung tumorigenesis by deleting Lmo4 in mice expressing the oncogenic K-Ras^G12D^. Our results showed that in the context of naphthalene-induced sensitization of K-Ras^G12D^-driven carcinogenesis, loss of Lmo4 reduced cell proliferation and delayed the onset of transformation but did not affect overall survival or tumor latency.

## Methods

### Mouse strains

*K-Ras*^*LSL-G12D*^ mice [28] and *Shh-Cre* mice [29] were purchased from the Jackson laboratory. *Lmo4*^*fl/fl*^ mice were obtained from Prof Visvader (The Walter and Eliza Hall Institute, Australia). *Scgb1a1-cre*^*ER*^ mice were a kind gift from Prof Hogan (Duke University) [30]. The *Lmo4*^*fl/fl*^ and *Lmo4*^*LacZ KI/+*^ mice have been described by Hahm *et al.* [11]. All animal experiments were conducted according to the Melbourne Health Research Directorate Animal Ethics Committee guidelines. Mouse tail DNA were genotyped by PCR using the following primers: Lmo4-floxed: 5′-CGAGCTGCTGCCCGGATTCAC-3′, 5′-GCATTCACCAGCCACAGATAAG-3′ and 5′-CGAGCTGAAATTGTCAGCAGCAAG-3′; *Shh-cre*: 5′-TTCGGCTATACGTAACAGGG-3′, 5′-TCGATGCAACGAGTGATGAG-3′; *K-Ras*^*LSL-G12D*^: 5′-CGCAGACTGTAGAGCAGCG-3′, 5′-CCATGGCTTGAGTAAGTCTGC-3′.

### Mouse treatment

For bromodeoxyuridine (BrdU) immunodetection, mice were injected with 0.5 mg per 10 g body weight 5-BrdU cell labeling reagent (Amersham Biosciences) 1 h before tissue collection. Naphthalene was dissolved in corn oil and injected i.p. at 250 mg/kg. H1N1 virus (PR8 mouse adapted virus; provided by A/Prof Belz, The Walter and Eliza Hall Institute, Australia) was resuspended at 10 PFU in 25μL of Dulbecco’s Phosphate-Buffered Saline (DPBS, Gibco) and administered by intranasal (i.n.) instillation. Adenovirus Ad5-CMV-Cre (University of Iowa Gene Transfer Vector Core) was resuspended in DPBS (10^10^ PFU/ml) and was administered intra nasally (i.n.; 20μL per mouse).

### Antibodies

For immunohistochemistry and immunofluorescence staining, the antibodies were α-LMO4 (clone 20F8, 7μg/ml), α-BrdU (Accurate Chemical and Scientific Corporation, OBT0030), α-keratin-5 (Covance, PRB-160P), α-Ki-67 (Cell Signaling, D3B5), α-p63 (Biocare Medical, CM163), α-CC10 (kind gift from Barry Stripp, Cedars-Sinai, CA), α-FoxJ1 (e-Bioscience, 14-9965), α-Nkx2.1 (Dako, M3575). Secondary antibodies were anti-rat biotin (Vector), anti-rabbit Alexa 594, anti-goat Alexa 488, anti-mouse Alexa 488 (Molecular Probes). Immunofluorescence stainings were imaged using DeltaVision Elite widefield microscope. Human sections were stained with α-TTF1 (DAKO, clone 8G7G3/1) or α-p63 (Millipore, MAB4135) antibodies. For Western blotting, α-LMO4 (clone 20F8) and α-GAPDH (Sigma-Aldrich, 71.1) antibodies were used.

### Histology and staining

For histological examination, lungs were fixed in 4 % paraformaldehyde in phosphate-buffered saline (PBS), embedded in paraffin, sectioned (2 μm) and stained with haematoxylin and eosin. For immunohistochemistry, sections were blocked in 10 % serum prior to incubation with specific antibodies or an isotype-matched control anti-rat (Pharmingen) or anti-rabbit (Cell Signaling) antibody diluted in PBS, 0.1 % tween containing 5 % serum, followed by biotin-conjugated secondary anti-rat or anti-rabbit antibody (Vector). Signal was amplified using Vectastain Elite ABC Reagent (Vector) for 30 min followed by 3, 3′-diaminobenzidine (DAKO).

For β-galactosidase activity staining, sorted cells were cytospun and fixed in 2 % paraformaldehyde, 0.2 % glutaraldehyde, 0.8 % NP-40, 0.01 % Na-deoxycholate for 5min at room temperature. Slides were then washed in PBS and stained with β-galactosidase staining solution (5 mM K_3_Fe(CN)_6_, 5 mM K_4_Fe(CN)_6_, 2 mM MgCl_2_, 0.8 % NP-40, 0.01 % Na-deoxycholate, 1 mg/ml X-gal) overnight at 37 °C. Slides were then counterstained with Nuclear-Fast Red and coverslipped.

Images of BrdU stained lung sections from Naphthalene injury and K-Ras^G12D^ models were acquired on Aperio ScanScope AT Slide Scanner (Leica Biosystems) and analysed with FIJI image processing software (http://fiji.sc). For Naphthalene injury model, BrdU positive cells were scored manually on the scanned images and number of positive cells in each airway was normalised to the length of airway circumference. Number of BrdU positive cells in hyperplastic airways at the week 12 time point was quantified using a custom FIJI script based on colour deconvolution and thresholding. The number of BrdU positive cells was normalised to the area of the corresponding hyperplastic airway and statistical analysis was carried out at the level of individual airways. For quantification of proliferating cells in hyperplastic airways at day six time point, number of BrdU positive cells normalised to area was calculated as above and averaged for each mouse based on ten airways per mouse. Statistical analysis was then performed at the mouse level. Tumour areas in K-Ras^G12D^ model was measured manually on the scanned images. Measurements were log transformed and statistical analysis was carried out at the level of individual tumours.

### RNA isolation and Real Time PCR

Total RNA was extracted from lung tissue using the miRNeasy Mini Kit (Qiagen) according to manufacturer’s instructions. DNase treatment was performed using TURBO DNA-free Kit based on the company’s instructions. cDNA was generated using SuperScript III (Invitrogen) according to the manufacturer’s instructions. qPCR was performed using the Sensimix SYBR Hi-Rox kit (Bioline) and the following primers for *Lmo4*: forward, 5′- CATGTTCTACCTGCCGGAAT-3′ reverse: 5′-TCTGGTCTGGCAGTAGTGGA-3′. PCR was carried out in the Rotorgene RG-6000 and expression levels were normalized against the expression of *18S rRNA* using the following primers: forward, 5′-GTAACCCGTTGAACCCCATT-3′; and reverse 5′-CCATCCAATCGGTAGTAGCG-3′.

### Fluorescent Activated Cells Sorting (FACS)

For FACS analysis, lungs were digested in collagenase (1mg/ml collagenase in 3ml DPBS + 0.2g/L glucose per lung) at 37 °C for 60 min while shaking at 165 rpm, followed by red blood cell lysis with 0.64 % ammonium chloride at 37 °C for 3 min [31]. Cells were resuspended in blocking solution (anti-FcR and Rat IgG) and incubated on ice for 10 min. Cells were stained with conjugated antibodies CD31-PECy7, CD45-PECy7, EpCAM-APC, CD104-FITC and CD24-PE (Biolegend) as described previously [32]. Cells were then washed and resuspended in PI solution. Cells were sorted on an ARIA II (Beckton Dickinson).

### Tissue microarray analysis

Tissue microarrays (2 mm cores, 3 per patient) were created from 34 squamous cell carcinomas, 40 adenocarcinomas and 10 otherwise classified Non Small Cell Lung Cancer (NSCLC) including large cell carcinomas cases collected from patients who underwent lung cancer surgery. Samples were obtained via the Victorian Cancer Biobank (Melbourne Australia). This study has Ethical Committee approval (Walter and Eliza Hall Institute Human Research Ethic Committee number 10/04). The scoring system for intensity was as follows: 0 - no staining; one - weak staining; two - moderate staining; and three - strong staining; and the scoring system for percentage was as follows: 0, no cells staining positive for LMO4; 1 - ≤ 10 % cells staining positive; 2 - 11 % to 50 % positive cells; 3 - 51 % to 80 % positive cells; and 4 - ≥ 80 % positive cells. The final histoscore (range from 0 to 12) was obtained from the product of the nuclear staining intensity and percentage of stained cells and averaged between three separate tissue cores from each of the patients. Difference in LMO4 histoscores between the cancer types was tested using Wilcoxon rank sum test.

### Statistical analysis

All statistical analysis was carried out using R software [33]. Overall survival difference between genotypes was tested by log rank test. To determine survival difference between each pair of the genotypes, log rank test was carried out on all pair-wise comparisons and resulting *p* values were adjusted for multiple comparisons using Holm method.

Unless otherwise specified, difference between means was tested using one-way ANOVA after assuring that assumptions of normality and equality of variance were satisfied. In cases, where residual errors did not follow normal distribution, variables were log transformed. Where necessary, *p* values were adjusted for multiple testing using Tukey HSD method.

Differences in the number of BrdU positive cells in the airways following Naphthalene injury (in normal and K-Ras^G12D^ lungs) were analysed using Wilcoxon rank sum test to accommodate multiple zero values.

Gene expression data for lung adenocarcinomas and squamous cell carcinomas was obtained from the respective public RNA-seq datasets (IlluminaHiSeq_RNASeq) of TCGA Research Network (http://cancergenome.nih.gov, accessed on 18/07/2014) [34, 35]. Raw count data was filtered to exclude lowly expressed genes (average log counts per million (cpm) < 0) and normalised using TMM normalisation in edgeR [36]. Log cpm values for *LMO4* were calculated across the samples using normalised library sizes and difference in *LMO4* expression between the cancer types was tested using one-way ANOVA.

## Results

### Lung-specific Lmo4 conditional knock-out mice display normal lung development

**Fig. 1:**
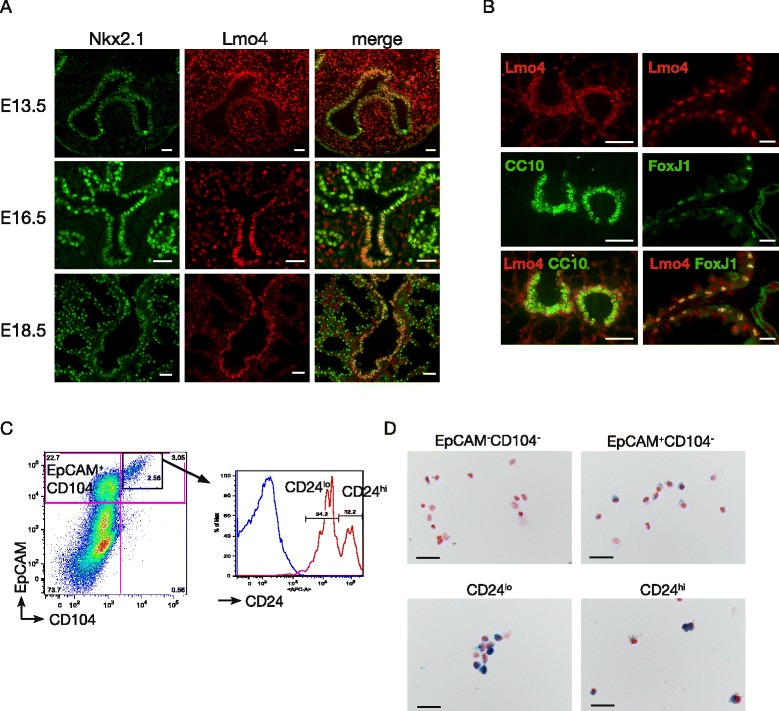
Lmo4 is expressed in lung epithelial cells. **a** Immunofluorescence staining for Nkx2.1 (green) and Lmo4 (red) in embryonic lung. Scale bars = 25 μm. **b** Immunofluorescence staining for CC10, FoxJ1 and Lmo4 in adult lung. Scale bars = 50 μm. **c** Representative FACS plots showing the sorting strategy to isolate adult lung epithelial subpopulations from live CD31^−^CD45^−^ cells. EpCAM^−^CD104^−^: non epithelial cells; EpCAM^+^CD104^+^ (also known as integrin β4): enriched in alveolar cells; EpCAM^+^CD104^+^CD24^lo^ (CD24^lo^): enriched in club cells and progenitor cells; EpCAM^+^CD104^+^CD24^hi^ (CD24^hi^): enriched in ciliated cells. **d** Representative photos of X-Gal and Nucleo-Fast Red counterstaining of sorted lung subpopulations isolated from *Lmo4* ^*LacZKI/+*^ mice. Scale bars = 50 μm

We first examined the expression of Lmo4 during embryonic lung morphogenesis, after birth and in adult. Quantitative RT-PCR results showed low levels of *Lmo4* expression in the early phase of embryonic development from E11.5 to E15.5. *Lmo4* expression peaks at E16.5 and then decreases in adult tissue (Additional file 1: Figure S1A). To further analyse the expression pattern of Lmo4 during embryonic lung development we performed immunofluorescence co-staining for Lmo4 and Nkx2.1, a marker of lung epithelial cells. Dynamic changes in the expression pattern of Lmo4 were observed (Fig. 1a). At E13.5, Lmo4 expression is detected in both the mesenchymal and endodermal compartments of the lung (Fig. 1a). At E16.5, strong Lmo4 expression is observed in the endoderm of the conducting airways while its expression is reduced in the distal branches from which the alveoli will form (Fig. 1a). At E18.5 the expression of Lmo4 becomes restricted to the cells lining the airways (Fig. 1a). Further evaluation of Lmo4 expression in adult lung shows that Lmo4 is expressed in club cells and ciliated cells, as shown by co-staining with CC10 and FoxJ1, markers of club cells and ciliated cells respectively (Fig. 1b). These results were confirmed by FACS analysis where lung epithelial cells were sorted based on the expression of EpCAM, CD104 (integrin β4) and CD24 (Heat Stable Antigen) as previously described by McQualter *et al.* [24] (Fig. 1c). Using these markers, EpCAM^+^ epithelial cells can be subdivided into CD104^+^ cells enriched in alveolar cells, CD104^+^CD24^lo^ cells enriched for club and progenitor cells and CD104^+^CD24^hi^ cells enriched in ciliated cells [24]. Using *Lmo4*^*LacZKi/+*^ mice in which the LacZ transgene has been knocked into the *Lmo4* locus [9] we observed β-galactosidase staining in CD104^+^CD24^lo^ and CD104^+^CD24^hi^ cells indicating that Lmo4 is expressed in CD104-positive cells (Fig. 1d).

To explore the role of Lmo4 in the lung epithelium, we generated *Shh-cre;Lmo4*^*fl/fl*^ mice in which *Lmo4* was excised from E9.5 in the epithelium of the lung primordia. Since the *cre* allele was knocked into the *Shh* locus resulting in loss of one *Shh* allele, *Shh-cre;Lmo4*^*fl/+*^ animals were used as controls. *Shh-cre;Lmo4*^*fl/fl*^ pups were born at normal mendelian ratio and survived to adulthood without any lung abnormalities compared to controls (Additional file 1: Figure S1B and data not shown), although complete excision of Lmo4 was observed in the epithelium of *Shh-cre;Lmo4*^*fl/fl*^ embryos (Additional file 1: Figure S1B). These results demonstrate that although Lmo4 is expressed in the embryonic lung, its role in the epithelium is not essential for normal lung morphogenesis.

### Loss of Lmo4 affects lung repair after H1N1-induced lung injury

**Fig. 2:**
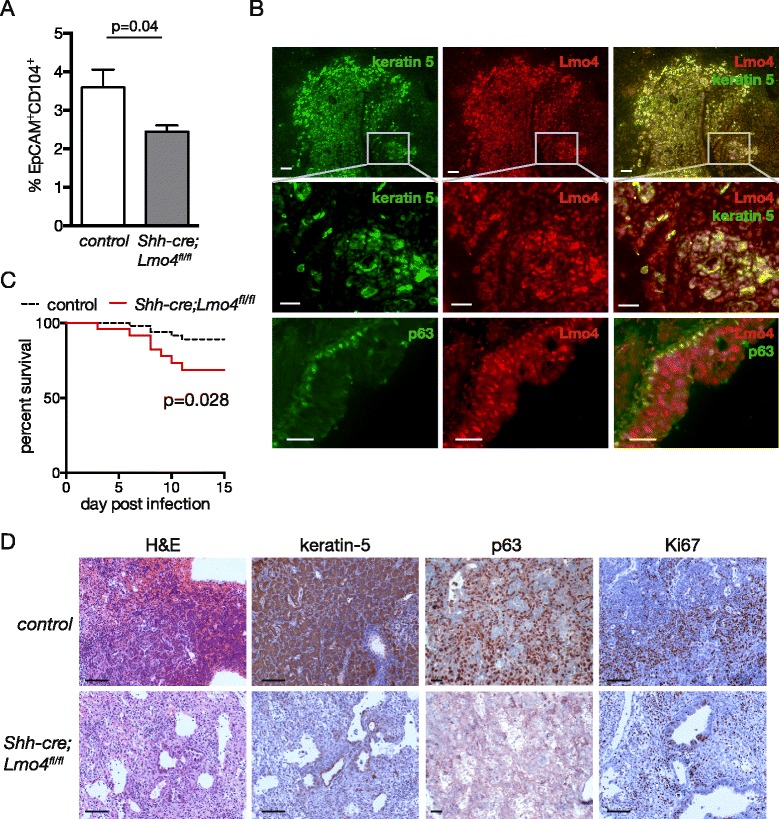
Loss of Lmo4 results in impaired lung repair following H1N1 infection. **a** Percentage of EpCAM^+^CD104^+^ cells in the lungs of naïve control or *Shh-cre;Lmo4* ^*fl/fl*^ animals. Data represent mean ± S.E.M., n = 5 animals per group. **b** Representative immunofluorescence staining showing the expression of Lmo4, keratin 5 and p63 in wild-type lungs 11 days post infection with H1N1 PR8 (10PFU). Scale bars = 25 μm**. c** Kaplan-Meier curve showing the survival of *Shh-cre;Lmo4* ^*fl/fl*^ animal (n = 27) compared to control animals (n = 69) following infection with PR8 flu virus. **d** Representative H&E and immunohistochemistry showing the expression of keratin 5, p63 and the proliferation marker Ki67 in control and *Shh-cre;Lmo4* ^*fl/fl*^ mice 11 days post infection with H1N1 (10PFU). Scale bars = 50 μm

Given that Lmo4 is expressed in CD104-positive cells that have recently been described to participate in the regeneration of the epithelium after flu-mediated lung injury [22], we investigated whether Lmo4 was required for adult lung progenitor cell function. We first observed that under steady state conditions, the number of CD104^+^ cells was reduced in *Shh-cre;Lmo4*^*fl/fl*^ compared to *Shh-cre;Lmo4*^*fl/+*^ control animals (Fig. 2a). However, when plated in colony formation assay on matrigel, the remaining CD104^+^ cells present in *Shh-cre;Lmo4*^*fl/fl*^ lungs had the same colony formation capacity as control animals (data not shown). These results suggested that loss of *Lmo4* does not affect CD104^+^ progenitor cells colony forming capacity *in vitro*.

To further explore the role of Lmo4 *in vivo* we infected *Shh-cre;Lmo4*^*fl/fl*^ and control mice with H1N1 flu virus. Intra-nasal administration of murine adapted (PR8) H1N1 influenza virus results in widespread bronchiolar and alveolar damage, with loss of club cells, ciliated cells and alveolar type 2 cells [20]. In the damaged areas, proliferation is maximal at 11 days post-infection and is driven by CD104-positive keratin 5-positive distal basal cells [20–22]. Interestingly, Lmo4 is expressed in these regions of active proliferation with keratin 5-positive cells expressing Lmo4 (Fig. 2b). Co-staining for Lmo4 and p63, another marker of basal cells, was also observed (Fig. 2b). *Shh-cre;Lmo4*^*fl/fl*^ mice showed an increased sensitivity to H1N1 infection with only 68 % of animals surviving to 11 days post infection compared to 89 % in the control group (Fig. 2c, log rank test *p* = 0.028, n ≥ 27). In both genotypes, animals that survived to 11 days recovered and returned to normal health within a few days. Interestingly, however, loss of Lmo4 was associated with a significant reduction in the percentage of keratin 5 expressing cells and Ki67-positive proliferating cells after H1N1 infection (Fig. 2d). Reduced animal recovery (68 %) and lower proliferation in Lmo4-depleted animals that survive the injury, suggest that Lmo4 is required for the proliferation of lung epithelial cells for the repair of the injured lung but is not essential for progenitor cells activity after flu-mediated injury.

### Loss of Lmo4 affects lung repair after naphthalene-induced lung injury

**Fig. 3:**
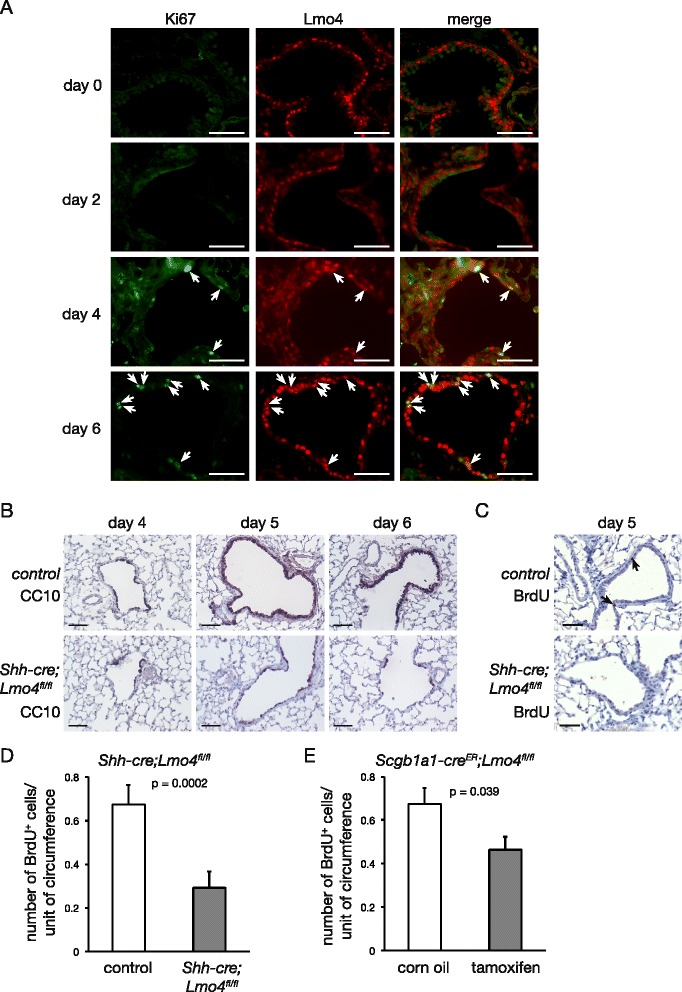
Loss of Lmo4 results in delayed repair after naphthalene-induced injury. **a** Representative immunofluorescence staining showing co-expression of Lmo4 (red) and Ki67 (green) in cells lining the airways of mice treated with naphthalene at the indicated time points. Scale bars = 50μm. White arrows indicate Ki67/Lmo4 double positive cells. **b** Immunohistochemistry showing the expression of the club cell marker CC10 in control and *Shh-cre;Lmo4* ^*fl/fl*^ lung sections 4, 5 and 6 days following naphthalene administration. Scale bars = 50μm. **c** BrdU staining of control and *Shh-cre;Lmo4* ^*fl/fl*^ lung sections five days following naphthalene administration. Scale bars = 50μm. Arrows indicate BrdU-positive cells. **d** Bar graph showing the number of BrdU-positive cells per airway circumference of control and *Shh-cre;Lmo4* ^*fl/fl*^ mice five days following naphthalene injury. Data represent mean ± S.E.M., n ≥ 39 airways per group from at least three animals per group. **e** Bar graph showing the number of BrdU-positive cells per airway circumference of control and *Scgb1a1-cre* ^*ER*^ *;Lmo4* ^*fl/fl*^ mice five days following naphthalene injury. Data represent mean ± S.E.M., n ≥ 47 airways per group from at least three animals per group. *p*-value computed by Wilcoxon rank sum test

Lmo4 expression in club cells in adult airways suggested that it might play an important role in repair after airway injury [37]. Treatment of mice with naphthalene damages the club cells lining the bronchiole with maximal injury of the airways observed two days following naphthalene treatment [26, 38]. Repair of the epithelium is driven by a population of variant club cells that are resistant to naphthalene-induced injury with newly regenerated epithelium observed one week after treatment [38]. In wild-type mice, although the majority of club cells are depleted two days after naphthalene injection, Lmo4 was detected in cells lining the airways at this time point (Fig. 3a). Interestingly, staining for the proliferation marker Ki67 revealed the presence of proliferative cells in the layer of flattened Lmo4-positive cells lining the basement membrane of the bronchiole four days following injury (Fig. 3a). Six days following injury, the number of proliferative cells increased and these cells co-expressed Lmo4 (Fig. 3a), suggesting that Lmo4 may play a role in lung epithelial cell proliferation after naphthalene injury. To explore the role of Lmo4 in airway repair following naphthalene injury, *Shh-cre;Lmo4*^*fl/fl*^ mice were treated with naphthalene and the lungs collected to analyse proliferation and airway repair. Lmo4 loss resulted in delayed repair with a reduction in the number of CC10-positive club cells five days post-naphthalene administration compared to control animals (Fig. 3b). BrdU immunostaining further showed a reduction in the number of proliferative cells in *Shh-cre;Lmo4*^*fl/fl*^ lungs compared to control (Fig. 3c and d, Wilcoxon rank sum test *p* = 0.0002). However, the animals survived the injury and eventually, the airways of *Shh-cre;Lmo4*^*fl/fl*^ mice returned to normal (data not shown). These results were confirmed using *Scgb1a1-cre*^*ER*^*;Lmo4*^*fl/fl*^ mice in which deletion of *Lmo4* occurs specifically in club cells after tamoxifen administration [30] (Additional file 2: Figure S2A). *Scgb1a1-cre*^*ER*^*;Lmo4*^*fl/fl*^ mice were first treated with corn oil or tamoxifen followed by naphthalene 2 weeks later. Analysis of BrdU incorporation in the lung five days after injury showed a significant reduction in the number of BrdU-positive cells in the airways of tamoxifen-treated mice (Fig. 3e, Wilcoxon rank sum test *p* = 0.039), consistent with the data observed in *Shh-cre;Lmo4*^*fl/fl*^ mice. These results suggest that Lmo4 promotes proliferation of epithelial cells after naphthalene-induced injury but is dispensable for complete repair of the airways.

### Loss of Lmo4 delays initiation of hyperplasia in *K-Ras*^*LSL-G12D/+*^ mice sensitized with naphthalene

**Fig. 4:**
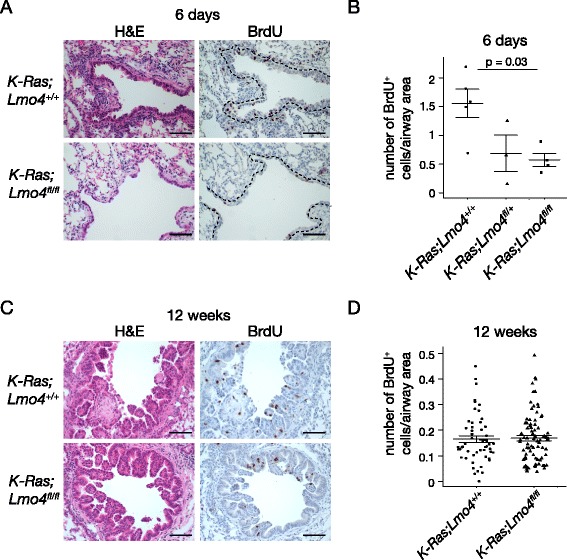
Loss of Lmo4 affects early stages of K-Ras^G12D^-induced cell proliferation. **a** H&E and BrdU staining of *K-Ras* ^*LSL-G12D/+*^ *;Lmo4* ^*fl/fl*^ and *K-Ras* ^*LSL-G12D/+*^ *Lmo4* ^*+/+*^ lungs six days following naphthalene injection. Scale bars = 100μm. Dashed line shows region used for quantification of BrdU staining. **b** Quantification of the number of BrdU^+^ cells per unit of airway area in *K-Ras* ^*LSL-G12D/+*^ *;Lmo4* ^*fl/fl*^, *K-Ras* ^*LSL-G12D/+*^ *;Lmo4* ^*fl/+*^ and *K-Ras* ^*LSL-G12D/+*^ *;Lmo4* ^*+/+*^ mice six days following naphthalene injection. n ≥ 3 animals per group. **c** H&E and BrdU staining of *K-Ras* ^*LSL-G12D/+*^ *;Lmo4* ^*fl/fl*^ and *K-Ras* ^*LSL-G12D/+*^ *Lmo4* ^*+/+*^ hyperplastic airways 11 weeks post naphthalene administration. Mice received Ad5-CMV-cre one week prior to naphthalene treatment. Scale bars = 100μm. **d** Quantification of the number of BrdU^+^ cells in *K-Ras* ^*LSL-G12D/+*^ *;Lmo4* ^*fl/fl*^ and *K-Ras* ^*LSL-G12D/+*^ *;Lmo4* ^*+/+*^ mice at 11 weeks post naphthalene administration. Mice received Ad5-CMV-cre one week prior to naphthalene treatment. Data represent number of BrdU^+^ cells per unit of airway area. The graph represents mean ± S.E.M., n ≥ 26 hyperplastic airways per group (n ≥ 3 animals per group)

Administration of naphthalene following K-Ras^G12D^ activation results in earlier tumor onset and increased tumor burden due to activation of progenitor cells in response to injury [27]. Given that Lmo4 is involved in the regulation of the proliferation of epithelial cells after naphthalene injury, we assessed whether loss of Lmo4 would affect initiation of tumor growth in naphthalene-mediated sensitization of K-Ras^G12D^-driven tumors. We used *K-Ras*^*LSL-G12D*^ model in which cre-mediated excision of the “STOP” cassette by intranasal administration of cre-expressing adenoviruses leads to activation of the oncogenic K-Ras^G12D^ in the lung [28, 39]. Upon Ad5-CMV-Cre infection, these mice initially develop adenomatous hyperplasia, which progresses to adenomas and adenocarcinomas [28]. *K-Ras*^*LSL-G12D/+*^*;Lmo4*^*fl/fl*^*, K-Ras*^*LSL-G12D/+*^*;Lmo4*^*fl/+*^ and *K-Ras*^*LSL-G12D/+*^*;Lmo4*^*+/+*^ mice were infected with Ad5-CMV-cre resulting in activation of K-Ras^G12D^ and excision of Lmo4 alleles in lung cells, followed one week later by naphthalene administration. Lungs were collected for analysis six days after naphthalene injection. At this time-point, areas of hyperplastic epithelium were observed around the airways of control animals but not in *K-Ras*^*LSL-G12D/+*^*;Lmo4*^*fl/fl*^ mice (Fig. 4a). Consistently, there was a marked decrease in the number of BrdU-positive cells in *K-Ras*^*LSL-G12D/+*^*;Lmo4*^*fl/fl*^ compared to *K-Ras*^*LSL-G12D/+*^ mice (Fig. 4a and b, one-way ANOVA *p* = 0.03, n ≥ 3). However, when lungs were collected 11 weeks following naphthalene injury (12 weeks following Ad5-CMV-cre administration), the number of proliferating cells in the hyperplastic airways was indistinguishable between the genotypes (Fig. 4c and d, Wilcoxon rank sum *p* = 0.5895). Lung weight (data not shown) and tumor area in the parenchyme (Additional file 2: Figure S2B) were also similar between the groups. These results indicate that loss of Lmo4 delays initiation of hyperplasia after naphthalene injury but this effect is rapidly overcome by signaling pathways activated by K-Ras^G12D^.

### Loss of Lmo4 does not affect tumor progression in K-Ras^G12D^-driven mouse model of lung cancer

**Fig. 5:**
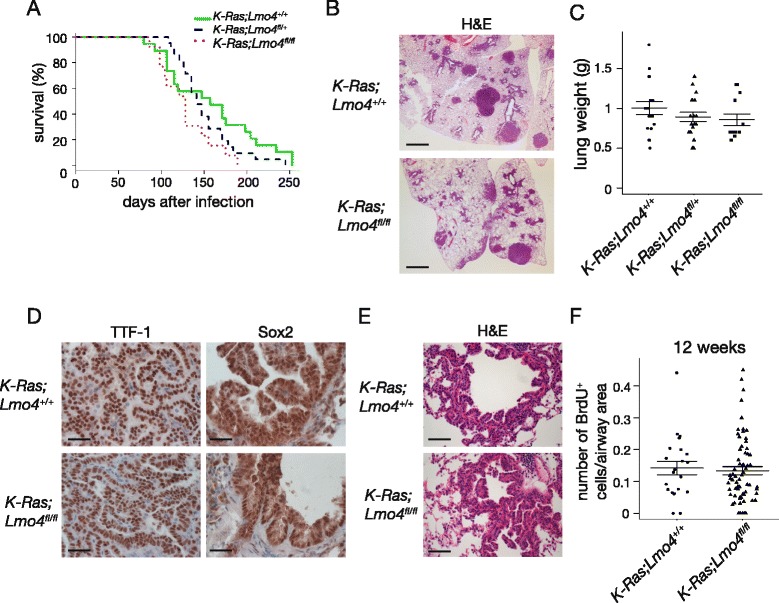
Loss of LMO4 does not alter overall survival in mouse model of lung cancer. **a** Kaplan-Meier curve showing overall survival of *K-Ras* ^*LSL-G12D/+*^ *;Lmo4* ^*fl/fl*^, *K-Ras* ^*LSL-G12D/+*^ *;Lmo4* ^*fl/+*^ and *K-Ras* ^*LSL-G12D/+*^ *;Lmo4* ^*+/+*^ mice following cre recombination with Ad5-CMV-cre adminstration. **b** Representative H&E showing overall tumour burden in *K-Ras* ^*LSL-G12D/+*^ *;Lmo4* ^*fl/fl*^ and *K-Ras* ^*LSL-G12D/+*^ *;Lmo4* ^*+/+*^ mice. Scale bar = 2mm. **c** Average lung weight of *K-Ras* ^*LSL-G12D/+*^ *;Lmo4* ^*fl/fl*^, *K-Ras* ^*LSL-G12D/+*^ *;Lmo4* ^*fl/+*^ and *K-Ras* ^*LSL-G12D/+*^ mice at ethical endpoint collection indicating overall tumor burden. The graph represents mean ± S.E.M., n ≥ 13 animals per group. **d** Immunohistochemistry showing the expression of TTF-1 and Sox2 in *K-Ras* ^*LSL-G12D/+*^ *;Lmo4* ^*fl/fl*^ and *K-Ras* ^*LSL-G12D/+*^ *;Lmo4* ^*+/+*^ tumours. Scale bars = 50μm. **e** Representative H&E staining of *K-Ras* ^*LSL-G12D/+*^ *;Lmo4* ^*fl/fl*^ and *K-Ras* ^*LSL-G12D/+*^ *;Lmo4* ^*+/+*^ hyperplastic airways 12 weeks following intranasal administration of Ad5-CMV-cre. Scale bars = 100μm. **f** Quantification of the number of BrdU^+^ cells in hyperplastic airways of *K-Ras* ^*LSL-G12D/+*^ *;Lmo4* ^*fl/fl*^ and *K-Ras* ^*LSL-G12D/+*^ mice at 12 weeks post Ad5-CMV-cre administration. Data represent number of BrdU^+^ cells per unit of airway area. The graph represents mean ± S.E.M., n ≥ 18 hyperplastic airways per group (n ≥ 3 animals per group)

We then assessed whether Lmo4 participated in tumor growth in the *K-Ras*^*LSL-G12D*^ model in the absence of naphthalene sensitization. *K-Ras*^*LSL-G12D/+*^*, K-Ras*^*LSL-G12D/+*^*;Lmo4*^*fl/+*^ and *K-Ras*^*LSL-G12D/+*^*;Lmo4*^*fl/fl*^ mice were infected intra-nasally with Ad5-CMV-cre resulting in activation of *K-Ras*^*G12D*^ expression and excision of *Lmo4* alleles in lung cells. Survival analysis showed no significant difference in the survival of *K-Ras*^*LSL-G12D/+*^*;Lmo4*^*fl/fl*^ mice (median survival 128 days, n = 13), when compared with *K-Ras*^*LSL-G12D/+*^*;Lmo4*^*fl/+*^ (median survival 141 days n = 22) and *K-Ras*^*LSL-G12D/+*^ (median survival 157 days, n = 20) control animals (Fig. 5a, log rank test *p* = 0.06). Histological examination and analysis of lung weight upon collection at ethical endpoint showed no significant difference in tumor burden in Lmo4-depleted lungs compared to control mice (Fig. 5b and c, one-way ANOVA *p* = 0.33, n ≥ 13), although Lmo4 expression was reduced in the tumors as assessed by Western Blot (Additional file 2: Figure S2C). All tumors presented histological features of adenocarcinomas and detailed morphological analysis did not show any significant difference in tumor grade, number of mitosis, presence of atypical adenomatous hyperplasia or bronchial hyperplasia between any of the genotypes (data not shown). Immunostaining for TTF-1, a marker of adenocarcinoma, and Sox2, a marker present in early lesions originating from CC10-positive cells and lost in advanced adenocarcinoma [40], revealed no difference between *K-Ras*^*LSL-G12D/+*^*;Lmo4*^*fl/fl*^ and *K-Ras*^*LSL-G12D/+*^ mice (Fig. 5d).

To evaluate whether loss of Lmo4 could affect tumour initiation, we collected mice of each genotype 12 weeks after Ad5-CMV-cre administration. Given that Lmo4 is essentially expressed in the airways, we quantified the number of BrdU-positive cells in hyperplastic airways and found no difference between Lmo4-depleted and control lungs (Fig. 5e and f, Wilcoxon rank sum *p* = 0.2773). Analysis of the lung weight (data not shown) or tumor area (Additional file 2: Figure S2D) did not show any significant difference between the cohorts. These results indicate that Lmo4 is not essential for tumor cell proliferation and overall tumor growth in the K-Ras^G12D^-induced mouse model of lung cancer.

### LMO4 is expressed in human lung squamous cell carcinoma

**Fig. 6:**
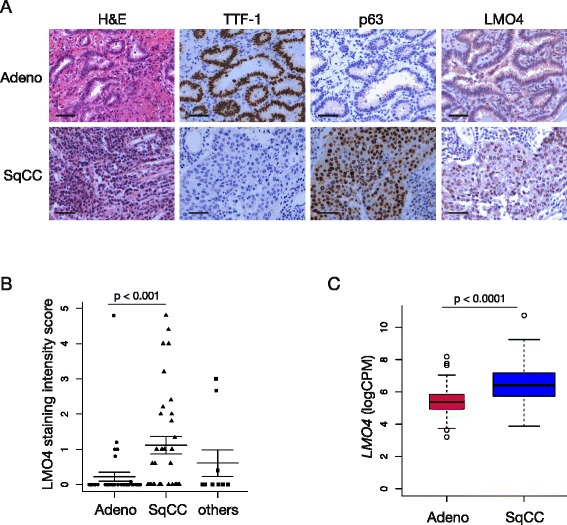
LMO4 is expressed in early stage lung squamous cell carcinoma. **a** Representative immunohistochemistry images showing the expression of TTF-1, p63 and LMO4 in human NSCLC cancer tissue microarray (TMA). Scale bars = 50μm. **b** Plot showing the histoscore for LMO4 expression in TMA for adenocarcinomas and squamous cell carcinomas (mean ± S.E.M.). **c** Box plot showing LMO4 expression levels (LogCPM: log count per million) in lung adenocarcinoma (Adeno) and squamous cell carcinoma (SqCC) RNAseq data from TCGA

Gene expression profiling analysis of lung tumors showed that LMO4 is highly expressed in advanced adenocarcinomas, which prompted us to analyse its role in K-Ras^G12D^ mouse model of adenocarcinoma [15]. As we did not observe any effect of Lmo4 loss on tumor-free survival in this model, we decided to further determine LMO4 expression in the different subtypes of NSCLC in early stage disease. We performed immunostaining for LMO4 in NSCLC tissue-microarray (TMA) of lung cancer samples obtained from patients undergoing lung cancer surgery (stage I-II disease) comprising 34 squamous cell carcinomas, 40 adenocarcinomas and ten otherwise-classified NSCLCs including large cell carcinomas. Scoring of LMO4 expression showed that LMO4 was significantly more expressed in squamous cell carcinomas compared to other tumor types (Fig. 6a and b, Wilcoxon rank sum test, *p* < 0.001). Consistently, analysis of The Cancer Genome Atlas (TCGA) data showed an increase in *LMO4* expression in lung squamous cell carcinomas (n = 224) compared to adenocarcinomas (n = 125) (Fig. 6c, one-way ANOVA *p* < 0.0001). These results suggest that LMO4 may play a more prominent role in the pathology of early stage squamous cell carcinoma than in adenocarcinomas. Depletion of Lmo4 in a mouse model of lung squamous cell carcinoma would enable to further explore this hypothesis.

## Discussion

Identifying factors regulating lung progenitor cell activity following lung injury is instrumental to deciphering processes driving lung repair. With their distinct properties to damage specific cell subtypes and to activate different progenitor cells, H1N1 infection and naphthalene treatment constitute complementary approaches to evaluate the role of particular proteins in the regulation of lung repair. Our results show that Lmo4 participates in the regulation of lung epithelial cell proliferation in these two models of injury. Lmo4 is dispensable for epithelial repair and animal survival but its loss delays recovery and increases sensitivity to H1N1-mediated injury. We also show that Lmo4 participates in the onset of lung cell proliferation induced by naphthalene in the context of oncogenic K-Ras^G12D^, further suggesting that Lmo4 plays an important role in lung epithelial cell proliferation.

Lmo4 is expressed in the developing lung with its expression becoming restricted to the airway lineages during development. This pattern of expression of Lmo4 in the embryonic lung is reminiscent of the expression profile of the transcription factor Sox2 where Sox2 is expressed only in the proximal airways and is absent in the distal tips of the branching epithelium that expresses Sox9 [41, 42]. Similar to Sox2, the expression pattern of Lmo4 becomes restricted to club cells and ciliated cells in the adult lung and is absent in the alveoli [42]. Given that half of *Lmo4*^*-/-*^ mice present breathing difficulties and die shortly after birth [11] we investigated the role of Lmo4 during lung morphogenesis by specifically ablating its expression in the lung epithelium from E9.5. No lung defects were observed in the conditional knock-out animals and their survival to adulthood was similar to their littermate controls. We observed that Lmo4 is expressed in both the epithelium and the mesenchyme until E13.5 before becoming restricted to the epithelium. Deletion of Lmo4 specifically in the mesenchyme would be required to evaluate whether Lmo4 plays a critical role in the mesenchyme during early lung morphogenesis.

Lmo4 has been implicated in the proliferation of progenitor cells in normal neural crest development where knockdown of Lmo4 leads to loss of neural crest precursor formation at the neural plate border [5] prompting us to evaluate the role of Lmo4 in lung progenitor cell activity. Repair of the adult lung is mediated by different progenitor cells depending on the type of injury inflicted. Following naphthalene administration, a rare population of variant club cells that do not express Cyp2f2 is maintained and responsible for the repopulation of the cells lining the airways [26, 30]. On the other hand, recent lineage tracing experiments showed that a rare population of keratin-5 positive basal cells present in the distal lung expand after flu injury to repair the lung [21] indicating that distinct progenitor cell populations are involved in the repair processes following naphthalene or H1N1 injury. Our results show that Lmo4 promotes epithelial cell proliferation in both models of lung injury. However, *in vitro* colony formation assay did not show any significant difference in the progenitor activity of Lmo4 conditional knockout cells compared to control cells, suggesting that Lmo4 may participate in cell proliferation but is not essential for lung progenitor cell function. Consistently, animals with Lmo4-depleted lungs manage to eventually recover from injury. Knock-down of Lmo4 has been shown to potentiate TGFβ signaling in HEK293 cells [10]. We previously showed that TGFβ inhibited lung epithelial cell colony formation capacity *in vitro* while SB431542, an inhibitor of TGFβ signaling, increased colony forming capacity *in vitro* [43]. Loss of Lmo4 in *Shh-cre;Lmo4*^*fl/fl*^ mice may therefore increase TGFβ signaling resulting in reduced epithelial cell proliferation. Other factors have been implicated in the proliferation of lung progenitor cells such as Bmi1 which regulates bronchio-alveolar stem cells (BASCs) expansion following naphthalene injury [44], p38α and PTEN which are involved in the expansion of BASCs [45, 46]. Stabilisation of β-catenin has also been shown to accelerate repair and increase CC10 expression three days following naphthalene injury [47]. Factors regulating distal basal cell expansion after H1N1 injury are still undefined. Lmo4 most likely cooperates with other regulatory factors to enable progenitor cells expansion and repair of the injured lung.

Recent studies have investigated the cell of origin of the different subtypes of lung cancer. Induction of oncogene expression or loss of tumor suppressor genes in specific cellular compartments using adenoviral delivery or genetically modified mouse models showed that neuroendocrine cells were the most likely cell of origin of small cell lung cancer [48] while both CC10-positive club cells and alveolar type II cells are involved in the initiation of tumor growth in *K-Ras*^*LSL-G12D/+*^*-* or *K-Ras*^*LSL-G12D/+*^*;p53*^*+/-*^*-*induced adenocarcinoma [40, 49]. In these models, expansion of CC10-positive cells after naphthalene injury has been described [40]. We observed that Lmo4 is expressed in club cells and is present in remaining proliferating cells lining the airways after naphthalene injury. Interestingly, Lmo4 deletion reduced cell proliferation after naphthalene injury in *K-Ras*^*LSL-G12D/+*^ mice, suggesting that upon K-Ras^G12D^ expression, Lmo4 is required for optimal proliferation of CC10-positive cells resistant to naphthalene injury. However this initial effect on cell proliferation is not observed 12 weeks following injury suggesting that Lmo4 is redundant for long-term Kras^G12D^-driven tumorigenesis. Using CMV-cre adenoviruses to activate K-Ras^G12D^ expression and deplete Lmo4 expression, we targeted all cell types in the lung, including alveolar type II cells that are the predominant cell of origin in K-Ras^G12D^-induced adenocarcinoma [40, 49] but do not express Lmo4. The lack of difference in overall survival between *K-Ras*^*LSL-G12D/+*^*;Lmo4*^*fl/fl*^ and *K-Ras*^*LSL-G12D/+*^ mice may therefore reflect the initiation of adenocarcinoma formation in alveolar type II cells. Deletion of Lmo4 and activation of K-Ras^G12D^ exclusively in CC10-positive cell would enable to resolve this hypothesis. However, given that the role of Lmo4 in progenitor cells is redundant, it is also likely that in the context of oncogenic transformation, Lmo4 is important in the early phase of transformation but is rapidly taken over by other factors controling transformed cell proliferation.

LMO4 is overexpressed in 50 % of breast cancer and 83 % of pancreatic adenocarcinoma [3, 50]. In lung cancer, gene expression analysis showed that *LMO4* is overexpressed in SCLC and advanced adenocarcinoma [15] but its expression has not been investigated in early stage disease in NSCLC. The association between LMO4 expression and patient outcome is still unclear. High LMO4 expression is associated with worse outcome in breast cancer [3] while in pancreatic adenocarcinoma, low LMO4 expression is associated with poor outcome [50]. In head and neck squamous cell carcinomas, LMO4 has been reported to be overexpressed at the invasive front of the tumor but its expression did not correlate with patient outcome [13, 51]. In a cohort of 84 NSCLC resected specimen from patients with stage I and II disease, we found that LMO4 expression was significantly higher in squamous cell carcinoma compared to adenocarcinoma. The size of the cohort in our current study did not enable us to evaluate correlation with patient outcome. Further investigations will be necessary to evaluate whether LMO4 is a predictor of outcome in lung squamous cell carcinoma and whether it plays a role in squamous cell carcinoma tumor progression. Lung squamous cell carcinoma is mostly observed in smokers. Our results show that loss of Lmo4 delays repair in acute models of lung damage such as naphthalene and H1N1 injury. It would be of interest to explore whether Lmo4 plays a prominent role in the repair of the lung in long-term injury models observed after exposure to oxidant such as cigarette smoke or ozone [52]. A role for Lmo4 in the regulation of cells proliferation in long-term injury may provide information regarding its role in the pathology of lung squamous cell carcinoma.

## Conclusions

Factors regulating lung repair following chemical or viral damage are still largely unknown. Here we show that the transcriptional regulator Lmo4 promotes lung epithelial cell proliferation and tissue repair following injury. Additionally, Lmo4 was found to modulate the early proliferative response observed after activation of oncogenic K-Ras^G12D^ expression in the context of naphthalene sensitization, although loss of Lmo4 did not affect overall tumor-free survival. This work provides novel insights into factors regulating lung repair following tissue damage caused by chemicals and pathogens present in the environment.

## Additional files

Additional file 1: Figure S1. Expression of Lmo4 in epithelial cells is not required for embryonic lung morphogenesis. **(A)** qRT-PCR analysis of *Lmo4* expression from E11.5 to E18.5, one week post-natal (Pn) and adult wild type lung (n = 4). Graph represents mean ± S.E.M. **(B)** Representative images showing immunohistochemistry for Lmo4 and Ki67 in sections of control and *Shh-cre;Lmo4* ^*fl/fl*^ embryonic lung. Scale bars = 50μm. Additional file 2: Figure S2. Lmo4 expression is lost in *Scgb1a1-creER;Lmo4* ^*fl/fl*^ mice and *K-Ras* ^*LSL-G12D/+*^ *;Lmo4* ^*fl/fl*^ mice. **(A)** Representative images showing immunohistochemistry for Lmo4 in sections of *Scgb1a1-creER;Lmo4* ^*fl/fl*^ mice treated with corn-oil or tamoxifen. Scale bars = 50μm. **(B)** Quantification of tumor size in *K-Ras* ^*LSL-G12D/+*^ *;Lmo4* ^*fl/fl*^, *K-Ras* ^*LSL-G12D/+*^ *;Lmo4* ^*fl/+*^ and *K-Ras* ^*LSL-G12D/+*^ mice at 11 weeks post naphthalene administration. Mice received Ad5-CMV-cre one week prior to naphthalene treatment. n ≥ 26 tumors per group from at least 3 animals per group. **(C)** Western blot analysis of Lmo4 expression in lung tumors isolated from *K-Ras* ^*LSL-G12D/+*^ *;Lmo4* ^*fl/fl*^, *K-Ras* ^*LSL-G12D/+*^ *;Lmo4* ^*fl/+*^ and *K-Ras* ^*LSL-G12D/+*^ mice. **(D)** Quantification of tumor size in *K-Ras* ^*LSL-G12D/+*^ *;Lmo4* ^*fl/fl*^, *K-Ras* ^*LSL-G12D/+*^ *;Lmo4* ^*fl/+*^ and *K-Ras* ^*LSL-G12D/+*^ mice at 12 weeks post administration of Ad5-CMV-cre. n ≥ 18 tumors per group from at least three animals per group. Graphs represent mean log tumour area ± S.E.M.

## Abbreviations

Adeno: Adenocarcinoma
Ad5: Adenovirus 5
ANOVA: Analysis of variance
BASCs: Bronchioalveolar stem cells
BrdU: Bromodeoxyuridine
CC10: Club Cell 10 protein
CMV: Cytomegalovirus
EpCAM: Epithelial Cell Adhesion Molecule
FACS: Fluorescent Activated Cells Sorting
GAPDH: Glyceraldehyde-3-Phosphate Dehydrogenase
K-Ras: Kirsten Rat Sarcoma viral oncogene homolog
NSCLC: Non-Small Cell Lung Cancer
LIM: Lin11, Isl-1 & Mec-3
LMO4: LIM-domain only 4
PBS: Phosphate-Buffered Saline
PTEN: Phosphatase and Tensin Homolog
Scgb1a1: Secretoglobin 1a1
SCLC: Small Cell Lung Cancer
Shh: Sonic Hedgehog
SqCC: Squamous Cell Carcinoma
TCGA: The Cancer Genome Atlas TCGA
TMA: Tissue Microarray
TTF1: Transcription Termination Factor RNA polymerase 1
