# Supplementary figures and images for: The LIM-domain only protein 4 contributes to lung epithelial cell proliferation but is not essential for tumor progression
Source: Respir Res. 2015 Jun 7;16(1):67. doi: 10.1186/s12931-015-0228-0 (PMC4475329; doi:10.1186/s12931-015-0228-0)

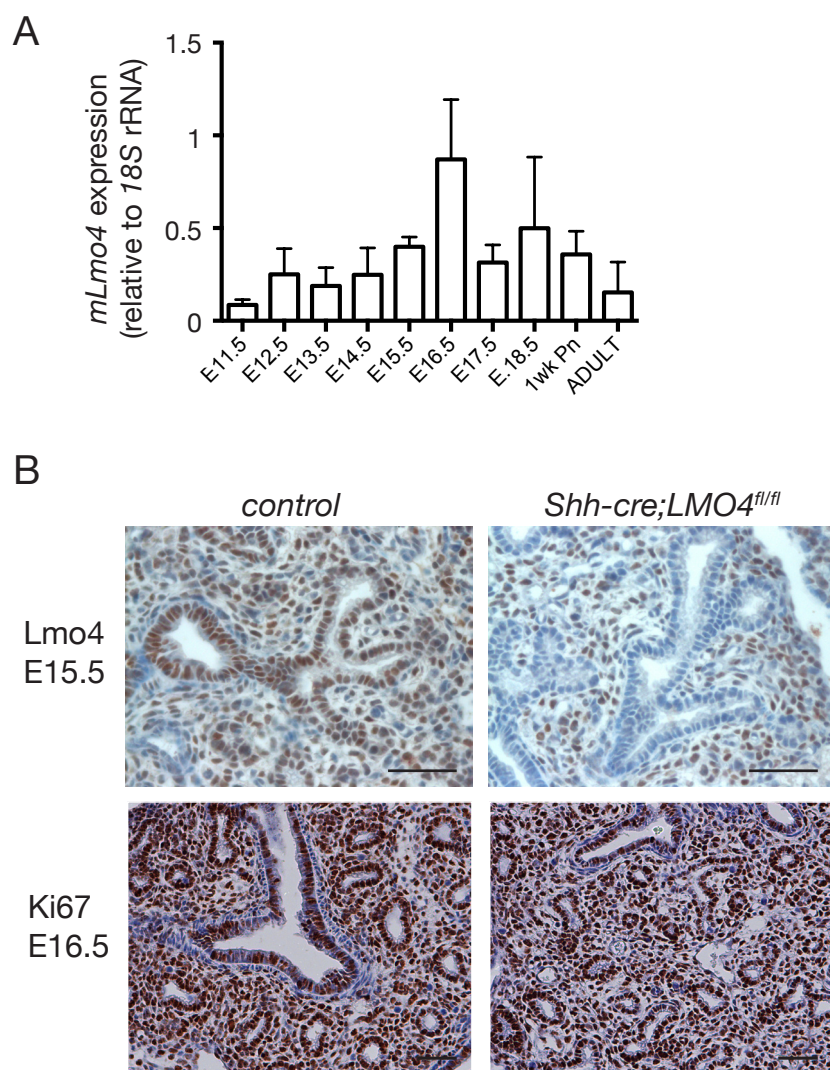

Supplement: Additional file 1: Figure S1. — Expression of Lmo4 in epithelial cells is not required for embryonic lung morphogenesis. (A) qRT-PCR analysis of Lmo4 expression from E11.5 to E18.5, one week post-natal (Pn) and adult wild type lung (n = 4). Graph represents mean ± S.E.M. (B) Representative images showing immunohistochemistry for Lmo4 and Ki67 in sections of control and Shh-cre;Lmo4 fl/fl embryonic lung. Scale bars = 50μm. [file 12931_2015_228_MOESM1_ESM.pdf]

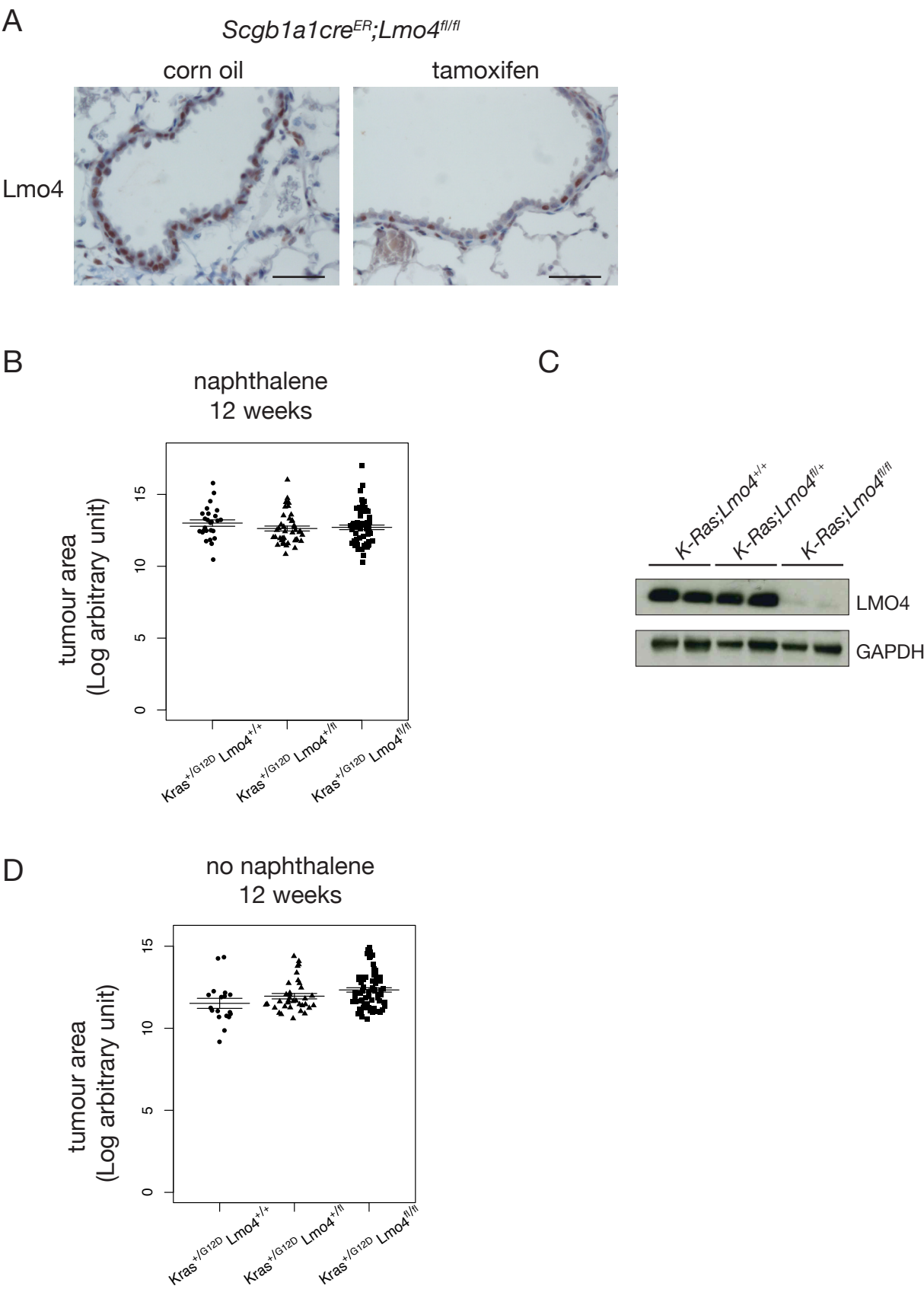

Supplement: Additional file 2: Figure S2. — Lmo4 expression is lost in Scgb1a1-creER;Lmo4 fl/fl mice and K-Ras LSL-G12D/+ ;Lmo4 fl/fl mice. (A) Representative images showing immunohistochemistry for Lmo4 in sections of Scgb1a1-creER;Lmo4 fl/fl mice treated with corn-oil or tamoxifen. Scale bars = 50μm. (B) Quantification of tumor size in K-Ras LSL-G12D/+ ;Lmo4 fl/fl, K-Ras LSL-G12D/+ ;Lmo4 fl/+ and K-Ras LSL-G12D/+ mice at 11 weeks post naphthalene administration. Mice received Ad5-CMV-cre one week prior to naphthalene treatment. n ≥ 26 tumors per group from at least 3 animals per group. (C) Western blot analysis of Lmo4 expression in lung tumors isolated from K-Ras LSL-G12D/+ ;Lmo4 fl/fl, K-Ras LSL-G12D/+ ;Lmo4 fl/+ and K-Ras LSL-G12D/+ mice. (D) Quantification of tumor size in K-Ras LSL-G12D/+ ;Lmo4 fl/fl, K-Ras LSL-G12D/+ ;Lmo4 fl/+ and K-Ras LSL-G12D/+ mice at 12 weeks post administration of Ad5-CMV-cre. n ≥ 18 tumors per group from at least three animals per group. Graphs represent mean log tumour area ± S.E.M. [file 12931_2015_228_MOESM2_ESM.pdf]
